# Fine Mapping and Candidate Gene Analysis of the Leaf-Color Gene *ygl-1* in Maize

**DOI:** 10.1371/journal.pone.0153962

**Published:** 2016-04-21

**Authors:** Haiying Guan, Xiangbo Xu, Chunmei He, Chunxiao Liu, Qiang Liu, Rui Dong, Tieshan Liu, Liming Wang

**Affiliations:** 1 Maize Research Institute, Shandong Academy of Agricultural Sciences, Jinan, China; 2 Key Laboratory of Biology and Genetic Improvement of North Summer Maize, Ministry of Agriculture, Jinan, China; 3 National Maize Improvement Sub-Center, Jinan, China; University of Tsukuba, JAPAN

## Abstract

A novel yellow-green leaf mutant *yellow-green leaf-1* (*ygl-1*) was isolated in self-pollinated progenies from the cross of maize inbred lines Ye478 and Yuanwu02. The mutant spontaneously showed yellow-green character throughout the lifespan. Meanwhile, the mutant reduced contents of chlorophyll and Car, arrested chloroplast development and lowered the capacity of photosynthesis compared with the wild-type Lx7226. Genetic analysis revealed that the mutant phenotype was controlled by a recessive nuclear gene. The *ygl-1* locus was initially mapped to an interval of about 0.86 Mb in bin 1.01 on the short arm of chromosome 1 using 231 yellow-green leaf individuals of an F_2_ segregating population from *ygl-1*/Lx7226. Utilizing four new polymorphic SSR markers, the *ygl-1* locus was narrowed down to a region of about 48 kb using 2930 and 2247 individuals of F_2_ and F_3_ mapping populations, respectively. Among the three predicted genes annotated within this 48 kb region, *GRMZM2G007441*, which was predicted to encode a cpSRP43 protein, had a 1-bp nucleotide deletion in the coding region of *ygl-1* resulting in a frame shift mutation. Semi-quantitative RT-PCR analysis revealed that *YGL-1* was constitutively expressed in all tested tissues and its expression level was not significantly affected in the *ygl-1* mutant from early to mature stages, while light intensity regulated its expression both in the *ygl-1* mutant and wild type seedlings. Furthermore, the mRNA levels of some genes involved in chloroplast development were affected in the six-week old *ygl-1* plants. These findings suggested that *YGL-1* plays an important role in chloroplast development of maize.

## Introduction

The color of plant leaves can significantly vary due to differences in the content of chlorophyll in the chloroplast. This type of mutation is known as chlorophyll deficient and is also called leaf color mutation. Such mutations have been reported in many higher plants since the 1930s, including rice [1], soybean [2], maize [3], barley [4], wheat [5], tomato [6], oilseed rape [7], and *Arabidopsis* [8].

Mutated genes for leaf-color influence chlorophyll biosynthesis and degradation as well as chloroplast development [9–12], and are ideal plant materials to investigate molecular mechanisms that regulate such pathways. The characterization of numerous genes in recent years has yielded major advancements in understanding mechanisms of leaf color mutation. For example, *elm1*, *elm2*, *vyl-Chr*.*1* and *vyl-Chr*.*9* in maize [13–15]; *ygl1*, *ygl2*, *ygl3*, *ygl7*, *ygl98* and *YGL138*(*t*) in rice [16–21]; and *chaos*, *egy1*, *apg1*, and *var3* in *Arabidopsis* [22–25] have been cloned and characterized. The pathway of chlorophyll biosynthesis is particularly well understood in *Arabidopsis*, in which 27 genes for all 15 steps and 15 kinds of enzymes in this role have been cloned and characterized [26–27]. Molecular mechanisms that regulate chlorophyll degradation and chloroplast development have been defined by major genes affecting these processes in *Arabidopsis* [28–29].

There are various leaf color mutants known in maize, such as albino, etiolated, light green, stripe or spot, virescent (v), and zebra according to their phenotypic characteristics [30]. According to the maizegdb database (http://www.maizegdb.org/), more than 210 leaf color mutant loci have been identified, distributed over all 10 maize chromosomes. Most genes associated with leaf color mutation are just approximately located to different chromosomes or chromosomal regions, with only a subset cloned and characterized. Among the cloned genes, three encode chlorophyll biosynthesis enzymes or their related proteins. *elm1* encodes a phytochromobilin synthase, and its seedling mutant plants have a pale green leaf, pronounced elongation of the mesocotyl, and disrupted de-etiolation responses to red and far-red light [31, 13]. Mature mutant plants maintain the pale green leaf phenotype and flower earlier than wild-type plants under long days [13]. *Oy1* encodes the I subunit of magnesium chelatase (ZmCHLI), and its seedling mutant plants exhibit yellow-green leaves that can be enhanced by growth at low temperature (24°C) [32]. Mature, field-grown mutant plants show a pale-green phenotype consistent with chlorophyll deficiency. *elm2* encodes a heme oxygenase (HO) that is required for chlorophyll biosynthesis, and its mutants spontaneously develop a yellowish phenotype during their entire life [14]. This gene was isolated by map-based cloning, and the 31 bp deletion in the coding region which reduced endogenous HO activity and disrupted the de-etiolation responses to red and far-red signal at seedling stage. Two genes involved in chloroplast development were also characterized. *hcf60* encodes the chloroplast ribosomal small subunit protein 17, and its mutants show temperature and light-dependent chlorophyll deficiencies [33]. *vyl* encodes the ClpP5 subunit of the chloroplast Clp protease, and its mutants exhibit a yellow leaf phenotype after emergence but can recover and become indistinguishable from wild-type plants after about 2 weeks [15].

The above-mentioned leaf-color mutant loci provided useful information in unraveling the chlorophyll synthesis and chloroplast development in maize. However, its mechanisms of leaf color mutation are still far from being fully understood and new genes related to leaf color mutation are of value. Among them, chlorophyll deficient mutants related to the chloroplast signal recognition particle (cpSRP) pathway could be useful tools for studying the progress of chloroplast development in maize [34–35]. The cpSRP contains four proteins that work together: cpSRP54, cpSRP43, cpFtsY, and ALB3 [36].

In this study, we isolated a new recessive yellow-green leaf mutant *ygl-1* in maize. The mutant spontaneously exhibited yellow-green leaves phenotype throughout all growth stages. By map-based cloning we isolated the *ygl-1* gene. It was predicted to encode a cpSRP43 protein and acts as a chloroplast precursor. Physiological and microscopic analysis revealed that chloroplast development was affected. Transcript levels of some genes associated with chloroplast development were significantly changed in *ygl-1* six-week old plants. Our findings provided direct evidence that the *YGL-1* gene plays an essential role in chloroplast development of maize.

## Materials and Methods

### Plant materials and mapping population

The *ygl-1* mutant was identified in self-pollinated progenies from the cross of two maize inbred lines Ye478 and Yuanwu02. An F_2_ segregating population was constructed from the cross of a *ygl-1* mutant and normal green leaf variety Lx7226. A total of 231 recessive yellow-green leaf individuals from three ears were selected and used for initial mapping. Normal green plants from the same three ears were self-pollinated to generate an F_3_ segregating population. A total of 2247 individuals from the F_3_ segregating population were used for fine mapping. Later, a large F_2_ population was obtained from a cross of the *ygl-1* mutant and normal green leaf variety Lx7226. To fine map *ygl-1*, 2930 individuals from the F_2_ population were screened. Plants with crossovers were self-pollinated to generate offspring for phenotypic verification. All the plants used for mapping *ygl-1*, chlorophyll content determination, transmission electron microscopy analysis and semi-quantitative RT-PCR were grown in the greenhouse of the Shandong Academy of Agricultural Sciences.

### Measurement of chlorophyll and carotenoid contents and photosynthetic characteristics

Leaf samples of wild type Lx7226 and the *ygl-1* mutant (of approximately 100mg fresh weight, respectively) were cut and submerged in 10ml of a 2:1 solution of 95% (V/V) acetone and ethanol for 48h at 26°C under dark conditions. The concentrations of total chlorophyll (total Chls), chlorophyll *a* (Chl *a*), chlorophyll *b* (Chl *b*), and carotenoids (Car) were measured with UH5300 UV/Vis spectrophotometer (Hitachi) at 663, 645, and 470nm, respectively. Pigment contents were calculated according to the following equations with slight modification obtained from the methods of Arnon [37].

Ch1a(mg/g)=[(12.7*OD663−2.69*OD645)*V]/(W*1000)

Ch1b(mg/g)=[(22.9*OD645−4.68*OD663)*V]/(W*1000)

Car(mg/g)=[OD470*(V/W)−3.27*Chla−104*Chlb]/198

Photosynthetic characteristics of wild-type Lx7226 and the *ygl-1* mutant were measured on sunny mornings from 9:00 to 10:00 h under greenhouse condition using a *Li-6400* portable photosynthesis system. The data were recorded at the middle of the ear leaves at tasseling stage.

### Transmission electron microscopy analysis

Leaf samples of wild type Lx7226 and the *ygl-1* mutant were harvested at different stages of development from the greenhouse. Only the top fully expanded leaves excluding the flag leaf were taken as samples. The fresh leaves were quickly cut into strips of 2mm width. The strips were fixed in a solution of 2.5% (v/v) glutaraldehyde and further fixed with 1% osmium tetroxide. The tissues were dehydrated through a series of acetone solutions and embedded in EMBed-812 prior to thin sectioning on Ultracut UCT (Leica). They were viewed using a transmission electron microscope H-7500 (Hitachi).

### DNA extraction

Leaf tissue of each plant was harvested at the two-week old stage, and genomic DNA was extracted by the CTAB method [38]. At the same time, 15 green and yellow-green plants each were randomly selected from the F_2_ population to construct DNA pools used to screen for polymorphic markers between *ygl-1* and Lx7226.

### Marker development and gene prediction

SSR markers used for linkage analysis were obtained from the Maize Genetics and Genomics Database (www.maizegdb.org). To fine map the region of the target locus, additional SSR markers were obtained from previous studies [39]. Gene prediction and annotation within the located region was conducted by Softberry and according to the Maize Genetics and Genomics Database.

### Gene cloning and sequence analysis

The DNA sequences of candidate gene *GRMZM2G007441* were amplified from both the *ygl-1* and its wild-type parents using primer pairs 21-3F (5′-CCCAAACGAACATGACCTAAAGC) and 21-6R (5′-TTCATCAAGTAATCTCTATCACCTGC). The target DNA fragments were cut from the gel and purified by the DNA Gel Extraction kit (TransGen Biotech). The fragments were connected to the pEASY-T1 cloning vector and sequenced at Invitrogen Biotech. The mutant site of the candidate gene was confirmed by the comparison and analysis of the sequencing results using DNAStar software. Multiple DNA sequence analysis was performed using DNAMAN version 5.0 software.

### Multiple amino acid sequence alignment and phylogenetic analysis

The full length protein sequence of *YGL-1* (*GRMZM2G007441*) was obtained from GenBank (http://www.ncbi.nlm.nih.gov). Its homologs in the *ygl-1* mutant and 6 wild type lines were obtained according to the translation to amino acid sequences from DNA sequences using BioXM 2.6 software. BLASTP searches against the nr database were carried out with the amino acid sequence of the maize gene *YGL-1* used as a query and its homologs from other species were retrieved from GenBank (http://www.ncbi.nlm.nih.gov). Multiple amino acid sequence alignment was conducted using DNAMAN version 5.0 software. Phylogenetic analysis was conducted by using MEGA version 5.1 with the rooted neighbor-joining tree using percentage identities based on a multiple sequence alignment generated with the DNAMAN 5.0 software.

### Semi-quantitative RT-PCR

Total RNA was isolated using an RNA Isolation Kit (Tiangen Biotech). High-quality first-strand cDNA was generated using oligo (dT) and PrimeScript™ II (Takara). Amplification of *ygl-1* and *YGL-1* cDNA (GenBank accession: DAA43190.1) was performed using primer pairs (21JF: 5′-CCCAGCATCCCGAACCCAA, 21JR: 5′-AGATGGCGTTGTGGGAGGG). Specific primers of 19 genes used for semi-quantitative RT-PCR were designed using primer 5.0 or selected from previous study [31]. The maize *actin* gene (*GRMZM2G126010*) was used as a control in the experiment. The primer pairs were designed using primer 5.0 and shown in S1 Table.

## Results

### Characterization of the *ygl-1* mutant

**Fig 1:**
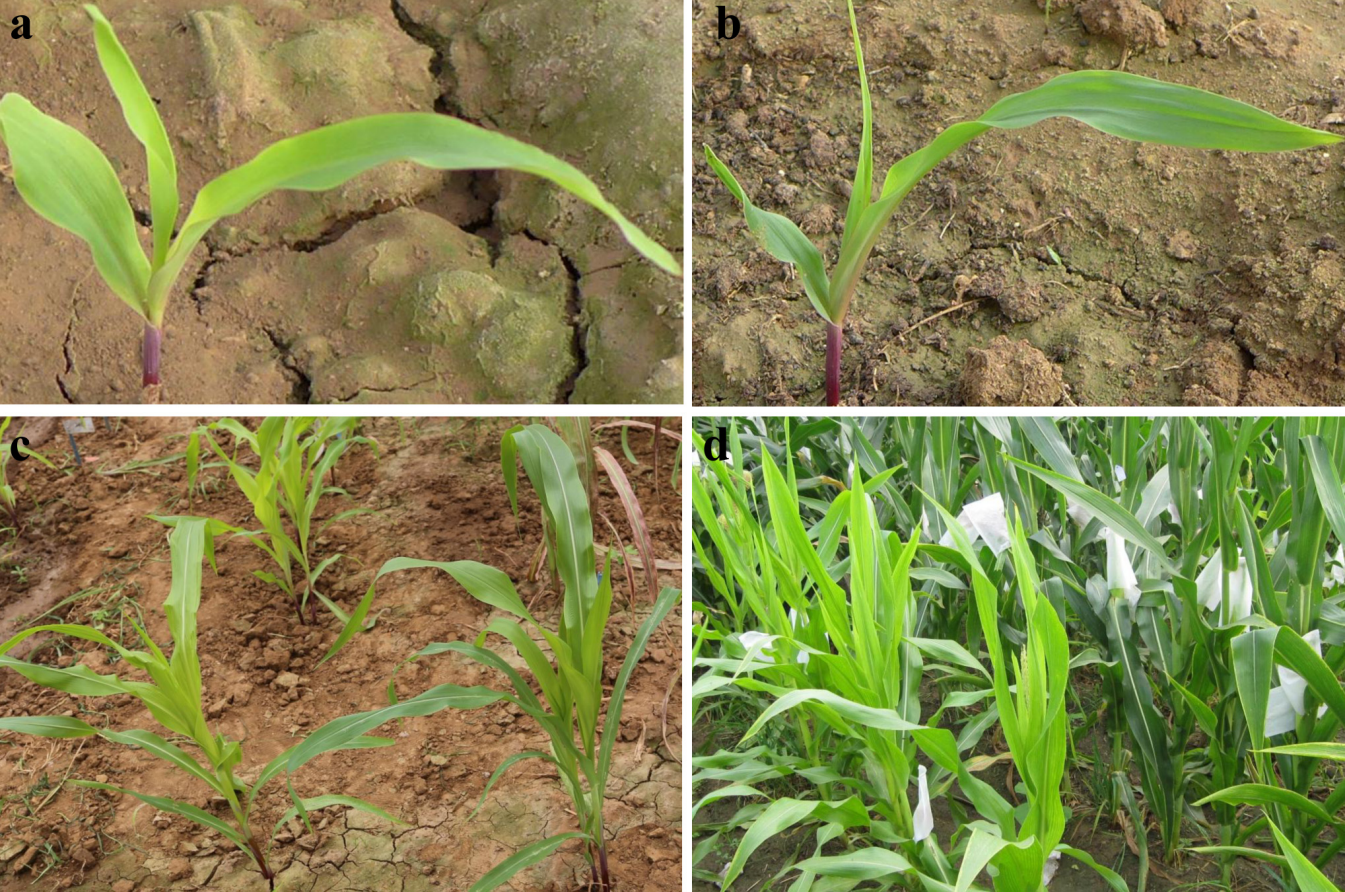
Phenotypic analysis of mutant *ygl-1* and wild type plants. (a, b), Phenotypic observation of 2-week old mutant plant *ygl-1* and wild type plant Lx7226. (c), Phenotypic observation of 6-week old mutant plants *ygl-1* (left) and wild-type plants Lx7226 (right). (d), Phenotypic observation of mutant plants *ygl-1* (left) and wild type plants Lx7226 (right) at tasseling.

A yellow-green leaf mutant, designated *ygl-1*, was a spontaneous mutant isolated in self-pollinated maize progenies from the cross of Ye478/Yuanwu02, and exhibited yellow-green leaves throughout development (Fig 1). All F_1_ plants of the *ygl-1* mutant crossed with wild-type inbred lines Lx7226, B73 and Chang7-2 displayed normal green leaves, and their F_2_ populations all showed a segregation ratio of 3:1 (green: yellow-green plants, χ2 < χ2_0.05_ = 3.84, P>0.05; Table 1). These results indicated that the yellow-green leaf phenotype in the *ygl-1* mutant was controlled by a single recessive nuclear gene.

**Table 1:** Segregation of F_1_ and F_2_ populations from three crosses.

Combination	F_1_ population	F_2_ population		ratio	χ2	P-value
		WT plants	*ygl-1* plants			
Lx7226 × *ygl-1*	green	218	78	2.79	0.29	0.5–0.7
B73 × *ygl-1*	green	212	62	3.41	0.82	0.3–0.5
Chang7-2 × *ygl-1*	green	205	63	3.25	0.32	0.5–0.7

WT, wild type.

To characterize the yellow-green leaf phenotype of *ygl-1*, the pigment contents of total Chls, Chl *a*, Chl *b*, and Car at seedling and tasseling stages were examined (Table 2). At the seedling stage, the contents of total Chls, Chl *a*, Chl *b*, and Car were 1.65, 1.39, 0.26, and 0.34 mg g^-1^ in the *ygl-1* mutant, significant reductions of 24.32%, 20.4%, 40.2%, and 1.96%, respectively, compared to the wild type Lx7226. At the tasseling stage, total Chls, Chl *a*, Chl *b*, and Car were further reduced to 48.43%, 35.71%, 72.79%, and 17.19% of wild type levels, respectively. At both seedling and tasseling stages, *ygl-1* mutants exhibited higher Chl *a*/*b* ratios than wild type, indicating that the reduction of Chl *b* was greater than Chl *a* in the *ygl-1* mutant. These results indicated that the *ygl-1* mutant phenotype was mainly because of reduced levels of total Chls, Car, and an increased ratio of Chl *a*/*b*. In addition, the significantly decreased content of Chl *b* in *ygl-1* might play an important role in its yellow-green leaf phenotype.

**Table 2:** Pigment contents in leaves of wild-type Lx7226 and *ygl-1* mutant, in mg g^-1^ fresh weight.

Lines	Total Chls	Chl *a*	Chl *b*	Chl *a*/*b* ratio	Car	Growth Stage
WT	2.18±0.02	1.75±0.00	0.43±0.00	4.06±0.01	0.35±0.00	seedling
*ygl-1*	1.65±0.01	1.39±0.00	0.26±0.00	5.40±0.01	0.34±0.00	seedling
Compared with WT	-24.32%**	-20.4%**	-40.2%**	33.11%**	-1.96%*	
WT	2.45±0.04	1.61±0.05	0.84±0.01	1.92±0.07	0.29±0.02	Tasseling
*ygl-1*	1.26±0.05	1.03±0.04	0.23±0.01	4.53±0.04	0.24±0.01	Tasseling
Compared with WT	-48.43%**	-35.71%**	-72.79%**	136.25%**	-17.19%*	

WT, wild type of Lx7226.

**, Significantly different at P≤0.01

*, Significantly different at P≤0.05.

**Fig 2:**
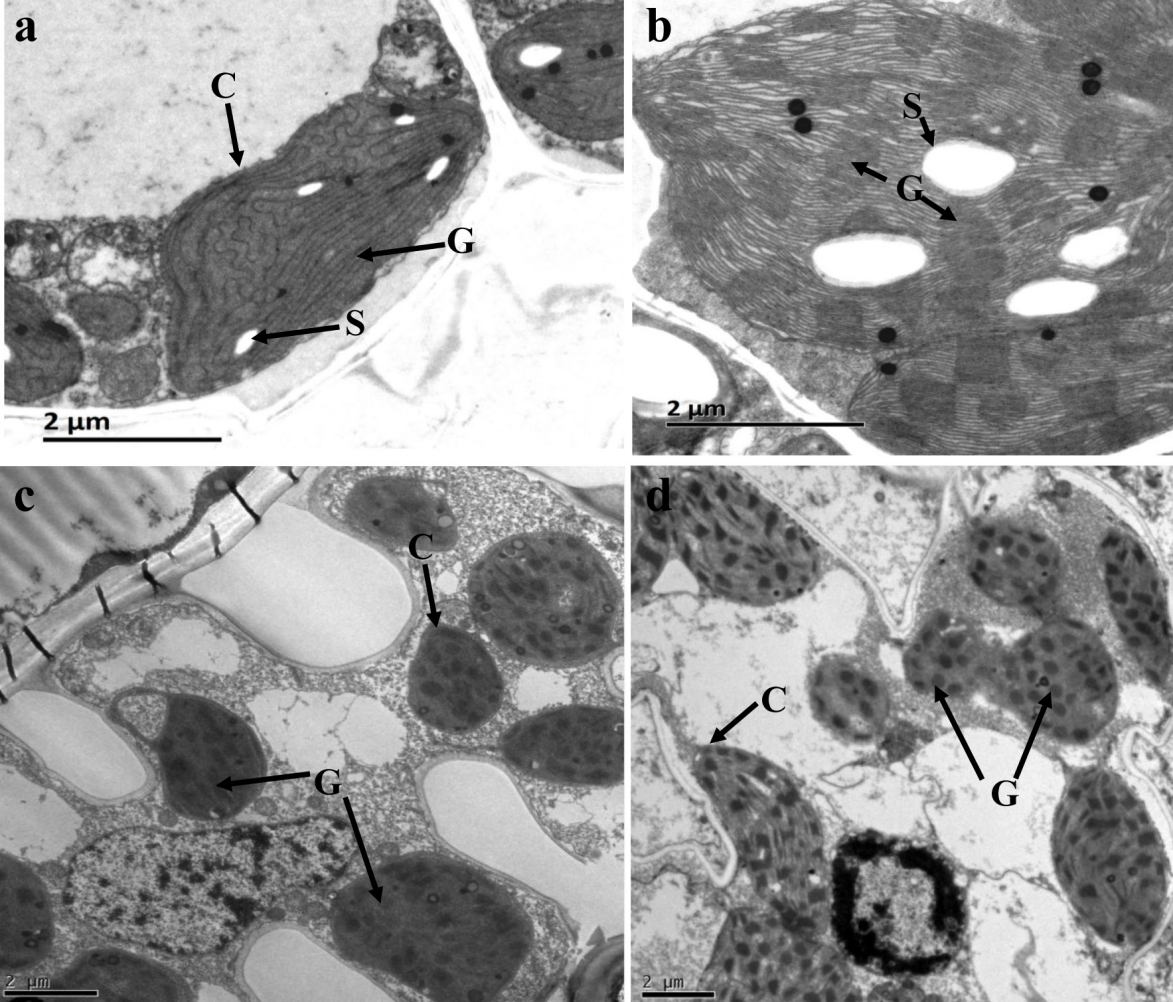
Transmission electron microscopy (TEM) analysis of chloroplast ultrastructure. (a, b), mutant plant *ygl-1* and wild type plant Lx7226 at the seedling stage, respectively. (c, d), mutant plant *ygl-1* and wild type plant Lx7226 at the tasseling stage, respectively. Bar equals 2 um. C, chloroplast; S, starch granule; G, granum.

To determine whether the yellow-green leaf phenotype of the *ygl-1* mutant affected chloroplast development, wild-type line Lx7226 and the *ygl-1* mutant leaf sections at the seedling and tasseling stages were compared using transmission electron microscopy. At the seedling stage, the *ygl-1* mutant had less non-appressed granal stacks (Fig 2A), while the wild-type inbred line Lx7226 had more and larger granal stacks (Fig 2B). Rare and less dense granal stacks appeared in the *ygl-1* mutant at tasseling (Fig 2C). The wild-type inbred line Lx7226 had more, well-ordered and dense granal stacks in the chloroplast (Fig 2D). However, there was no apparent difference in the structure of the chloroplast in the *ygl-1* mutant and wild-type. These results indicated that chloroplast development was suppressed in the *ygl-1* mutant.

We compared the photosynthetic characteristics between wild-type line Lx7226 and the *ygl-1* mutant at tasseling stage. As shown in Table 3, the net photosynthesis rate (*P*_n_), the stomatal conductance (*C*s), the intercellular CO_2_ concentration (*C*i) and the transpiration rate (*T*r) were significantly deceased in the *ygl-1* mutant compared with wild-type Lx7226. The results indicated that the capacity of photosynthesis was impaired in the *ygl-1* mutant, which might be consistent with its chlorophyll deficiency and impaired development of chloroplasts.

**Table 3:** Photosynthetic characteristics in the wild-type Lx7226 and *ygl-1* mutant at tasseling stage.

Materials	*P*_n_ (umol m^-2^s^-1^)	*C*s	*C*i (uL/L)	*T*r (mmol m^-2^s^-1^)
Lx7226	15.58±1.18	0.13±0.03	258.75±17.40	1.54±0.29
*ygl-1*	5.13±0.57**	0.05±0.01*	155.5±1.29**	0.76±0.11*

*P*_n_, net photosynthesis rate; *C*s, stomatal conductance; *C*i, intercellular CO_2_ concentration; *T*r, transpiration rate.

**, Significantly different at P≤0.01

*, Significantly different at P≤0.05.

### Initial mapping of *ygl-1*

**Fig 3:**
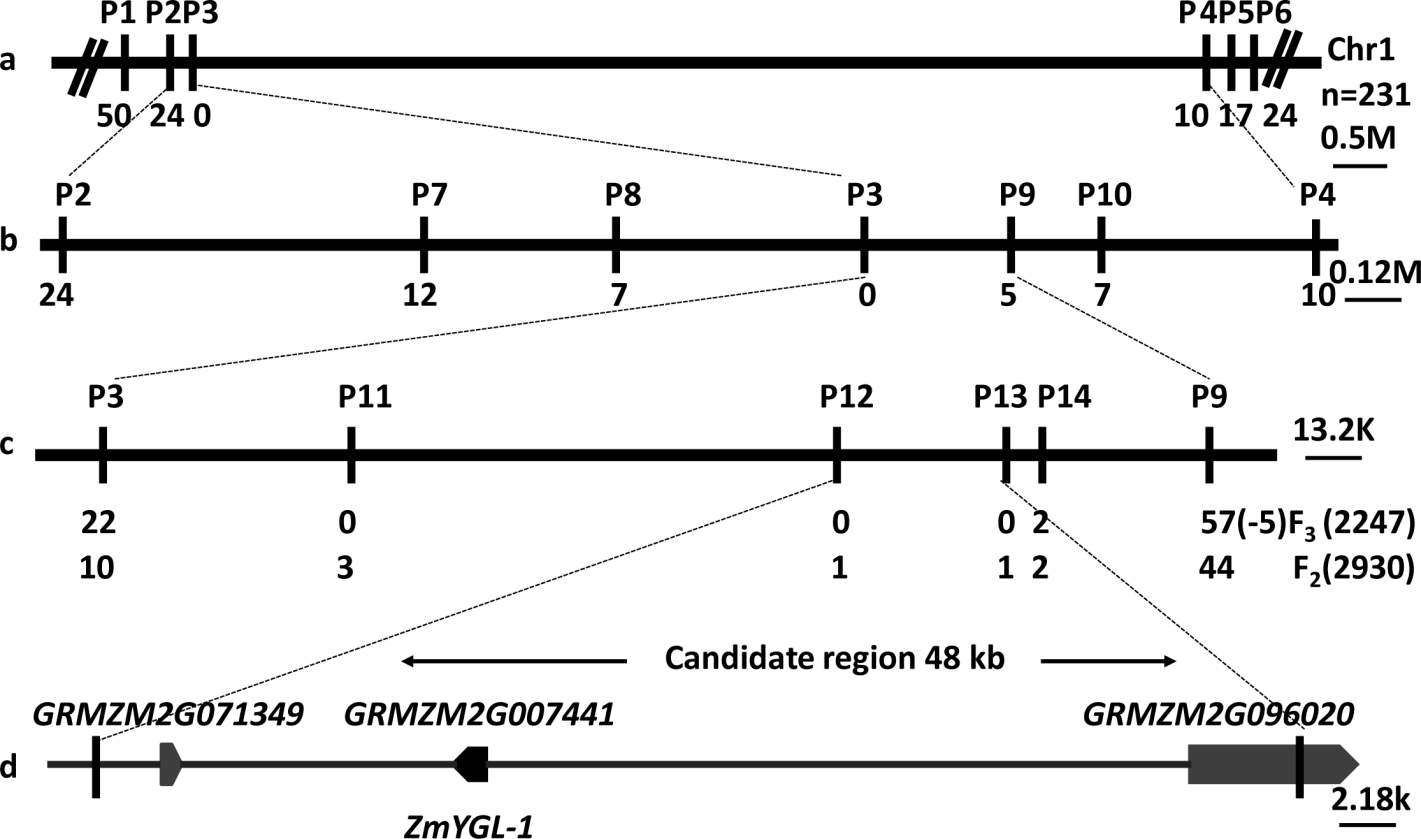
Fine mapping of the *ygl-1* locus. (a), The *ygl-1* locus was delimited to an interval flanked by markers P2 and P4 in bin 1.01 on the short arm of chromosome 1 using 231 yellow-green individuals of an F_2_ segregating population. (b), Utilizing 4 new polymorphic markers, *ygl-1* was mapped to a region between two markers P8 and P9. (c), The *ygl-1* locus was finally delimited to an interval of about 48 kb flanked by P12 and P13 using 2,247 individuals of F_3_ and 2,930 individuals of F_2_ populations, respectively. (d), There are three annotated genes in the candidate region according to the maizegdb database (www.maizegdb.org). The numbers below the horizontal lines represent recombinational events. The scale bars represent physical distance.

Preliminary mapping of *ygl-1* was conducted using 231 recessive individuals showing yellow-green leaf phenotype from an F_2_ segregating population obtained from the cross of *ygl-1*/Lx7226. A total of 224 pairs of SSR markers distributed over the 10 maize chromosomes, available from the public database maizegdb, were screened by polyacrylamide gel electrophoresis. We found only P3 in bin 1.01 on the short arm of this chromosome to exhibit polymorphism between the two parents and the DNA pools from the randomly selected 15 green and 15 yellow-green plants, respectively (see methods) (Table 4). A total of 23 SSR primers located in bin 1.01 available from maizegdb were detected by polyacrylamide gel electrophoresis between the two parents and the two DNA pools. Five of the 23 primers had polymorphisms both between the two parents and the two DNA pools. All six polymorphic markers were used to screen the 231 recessive individuals of F_2_. Finally, two of them, P1 and P2, were positioned on one side of *ygl-1* toward the telomeric end of chromosome 1, with 50 and 24 recombinants, respectively; the other three markers, P4, P5 and P6 were located on the other side of *ygl-1* toward the centromeric region, with 10, 17 and 24 recombinants, respectively (Table 4, Fig 3A). No recombinant was screened out by marker P3, suggesting that this marker was closely linked to *ygl-1*. These results indicated that the locus of *ygl-1* was between markers P2 and P4, which delineated an interval of 2.6 Mb according to the reported B73 whole genome sequence (Fig 3A). Four new SSR markers showing polymorphisms between two parents and two DNA pools were used to screen the 34 recombinant individuals detected by markers P2 and P4. A total of 12, 7, 5 and 7 plants with crossovers were found with P7, P8, P9 and P10, respectively. Thus, *ygl-1* was mapped to an interval of about 0.86 Mb between P8 and P9 (Table 4, Fig 3B).

**Table 4:** Markers used for genetic mapping of *ygl-1*.

BAC accession number	Marker	Primer sequence (5′-3′)	Product size (bp)
AC194148.5	P1	F:TCTTCATCTCTCTATCAAACTGACA	230
		R:TGGCACATCCACAAGAACAT	
AC193998.4	P2	F:GAAGTGGGGAACATGGTTAATGTC	157
		R:TCACGGTTCAGACAGATACAGCTC	
AC190890.6	P7*	F:TTGTCCCTGCTTGCATGACA	155
		R:TGGCTCGATCAACTTCCCTG	
AC193473.4	P8*	F:ACGAACAGGAGAACATGCGT	212
		R:CATGGCAGCCCACATTTGTT	
AC191330.4	P3	F:CGCCTGTGATTGCACTACAC	161
		R:CACGCTGTTTCAGACAGGAA	
AC191330.4	P11*	F:CTTCCCAAAAGCCACCCAGA	198
		R:GTGGATGCTTGCATGACGAC	
AC201968.5	P12*	F:CCTCAACTTCCCCATCTCCG	199
		R:TGCGCCTAACCTTCGAAGTT	
AC195193.4	P13*	F:CTCATTTTGTTCCAGACCCGC	144
		R:ACTGGTACCTTTCAGGGCAA	
AC195193.4	P14*	F:GTGCTGTGCATGCGTATCTG	204
		R:GTACCACCGACCATCCCATC	
AC195193.4	P9*	F:TTTCAGTTCGGCGTCGATCA	227
		R:GGGCCCGTGTACATGTTACA	
AC195884.4	P10*	F:GAGATCACCAGCCGTTCCTC	191
		R:GACGATAGGCGGTTCTCGTG	
AC190859.4	P4	F:TATATTAGAGGCACCTCCCTCCGT	377
		R:AGCTGCTTCAGCGACTTTGG	
AC190706.4	P5	F:GTGAGAATCCTTCAGCGGAG	182
		R:CTGTGGCAGATGTGGTATGG	
AC205622.1	P6	F:CGTTTGATATGATGTGGAGATTCG	135
		R:AAGCTTGTGAATGTTCTGGATGTC	

*, These eight SSR markers came from previous study [39].

### Fine mapping of *ygl-1*

To fine map *ygl-1*, four new SSR markers polymorphic between the two parents and two DNA pools were identified (Table 4). Meanwhile, 2247 and 2930 plants of F_3_ and F_2_ segregating populations, respectively, were screened to obtain more recombinant individuals. A total of 32 plants were confirmed with crossovers by P3 located on the side of P8, including 22 and 10 from F_3_ and F_2_ populations, respectively. A total of 96 recombinant plants were detected by P9, including 52 and 44 from F_3_ and F_2_ populations, respectively. All 133 (32 + 96 + 5) recombinant individuals screened by P3 and P9 were genotyped by new polymorphic SSR markers P11, P12, P13 and P14. Finally, only one recombinant was identified both by marker P12 and P13, on the opposite side of *ygl-1*. Thus, *ygl-1* was narrowed down to a genomic region of about 48 kb in the B73 genome sequence, flanked by markers P12 and P13 (Fig 3C).

### Candidate gene annotation and sequence analysis

**Fig 4:**
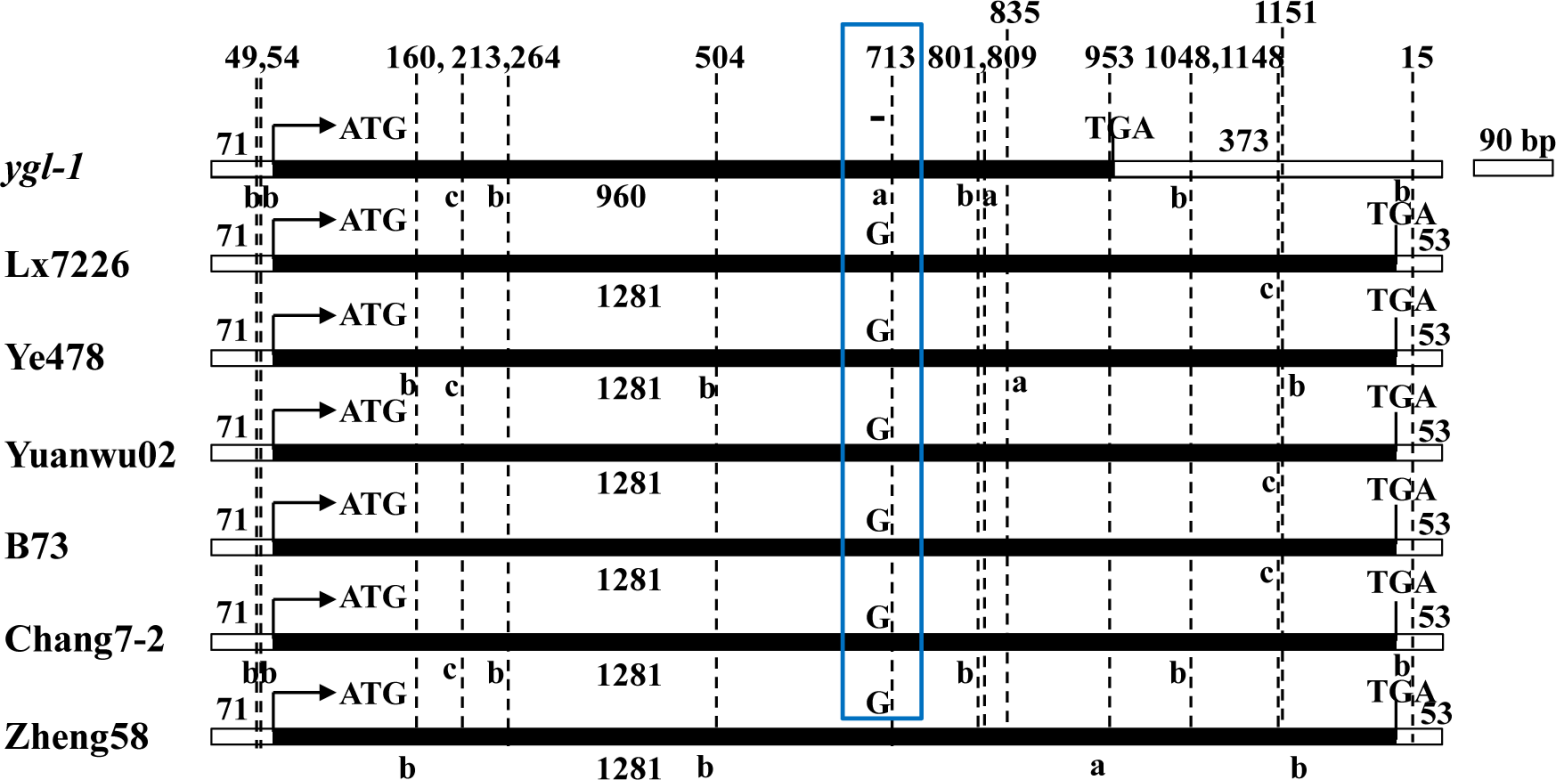
Schematic diagram of *YGL-1* genomic structure. Black boxes with numbers indicate ORFs; blank boxes with numbers indicate sequences upstream/downstream of the ORFs; light blue box indicates 1-bp (-/G) deletion at 713 bp position from the ATG start codon in *ygl-1*. a-c, a single SNP at specific position upstream/downstream or in the ORFs that can divide the seven lines into two different groups, which distinguish one material, two materials or three materials from other lines, respectively. Numbers above the dashed lines indicate the positions of SNPs that upstream, downstream or in the ORFs.

Gene prediction in the 48 kb region of the maize genome inferred to contain *ygl-1* revealed three genes (Fig 3D, S2 Table). One of the three genes, *GRMZM2G007441* comprises only one exon and was predicted to encode a signal recognition particle 43 kDa (SRP43) protein and act as a chloroplast precursor. This protein has been reported to confer a pale green leaf phenotype in *Arabidopsis* [22]. Motivated by these results, we sequenced the *GRMZM2G007441* gene from amplified genomic DNA of mutant line *ygl-1* and six wild-type inbred lines, Lx7226, Ye478, Yuanwu02, B73, Chang7-2 and Zheng58, using primer pairs 21-3F and 21-6R, respectively. A single-nucleotide was deleted at the position 713 bp (-/G) from the ATG start codon in *ygl-1*, which resulted in a frame shift mutation (Fig 4). The resulting 960 bp truncated translational region encodes a predicted protein of 319 amino acids in *ygl-1* compared to 1281 bp ORFs (open reading frame) encoding predicted proteins of 426 amino acids in all 6 wild-type lines (Fig 4 and S1 Fig). The conserved region of ANK_2 and ANK changed and CHROMO almost disappeared in *ygl-1* compared to the 6 wild-type lines (S1 Fig). To our knowledge, the other two candidate genes *GRMZM2G071349* and *GRMZM2G096020* were not reported to participate in Chl metabolism or chloroplast development (S2 Table).

**Fig 5:**
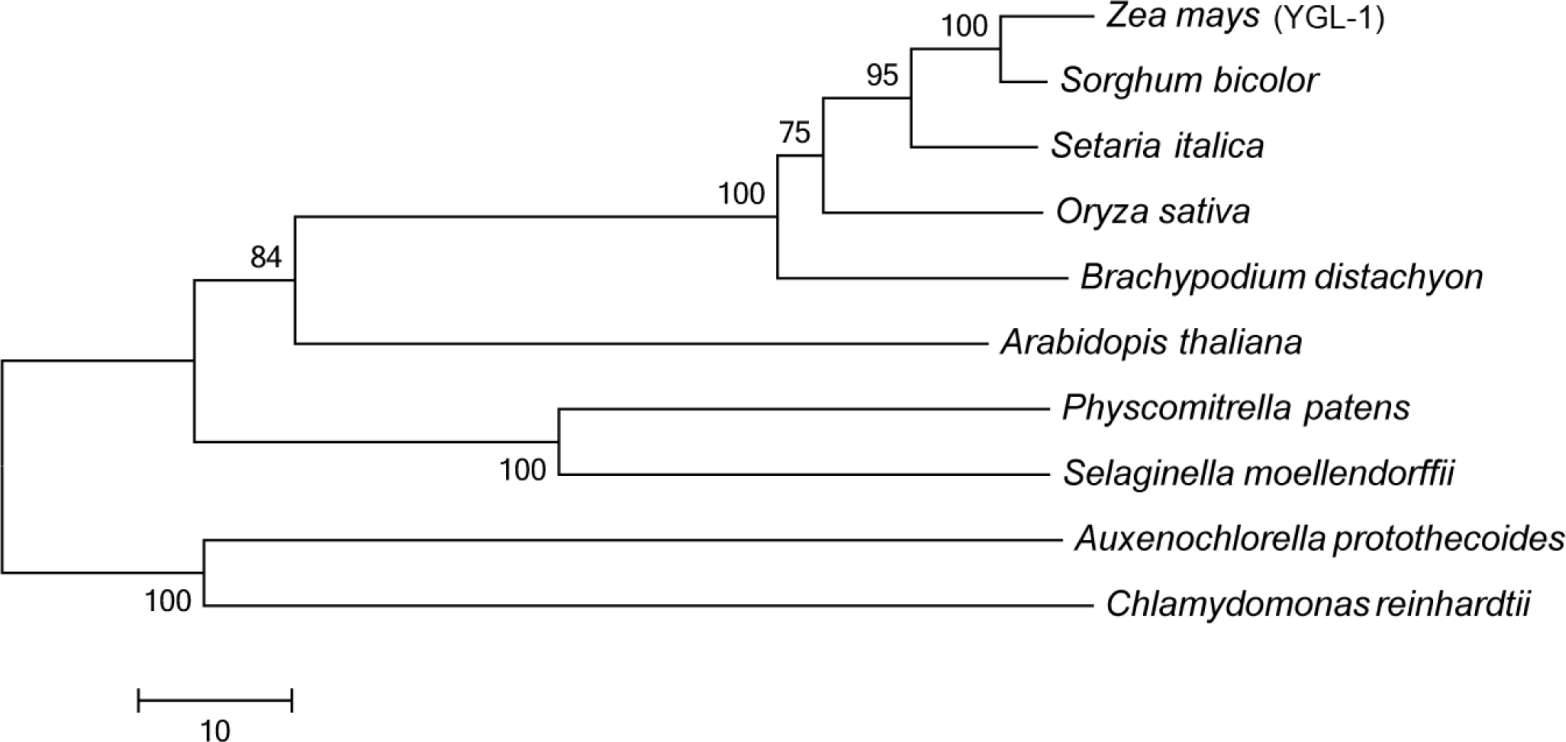
Phylogenetic anaysis of the YGL-1 protein and its related proteins. Accession numbers of the protein sequence were as follows: *Zea mays* [YGL-1, DAA43190.1]; *Sorghum bicolor* [XP_002465920.1]; *Setaria italica* [XP_004985832.1]; *Oryza sativa* [NP_001048866.1]; *Brachypodium distachyon* [XP_003562136.1]; *Arabidopsis thaliana* [NP_566101]; *Physcomitrella patens* [XP_001771412.1]; *Selaginella moellendorffii* [XP_002989974.1]; *Auxenochlorella protothecoides* [XP_011399198.1]; *Chlamydomonas reinhardtii* [AGC59877.1].

BLAST search of the maize genome database revealed that *YGL-1* (*GRMZM2G007441*) is a single-copy gene. We aligned the amino acid sequences of *YGL-1* and its related proteins from *Sorghum bicolor*, *Setaria italica*, *Oryza sativa* (LOC_Os03g03990), *Brachypodium distachyon*, *Arabidopsis thaliana* (AT2G47450), *Physcomitrella patens*, *Selaginella moellendorffii*, *Auxenochlorella protothecoides* and *Chlamydomonas reinhardtii*. YGL-1 exhibits most sequence similarity to the related protein in *Sorghum bicolor*, with 87% identity. YGL-1 also exhibits 81%, 76%, 71%, 54%, 47%, 46%, 43% and 36% sequence similarity to proteins in *Setaria italica*, *Oryza sativa*, *Brachypodium distachyon*, *Arabidopsis thaliana*, *Physcomitrella patens*, *Selaginella moellendorffii*, *Auxenochlorella protothecoides* and *Chlamydomonas reinhardtii*, respectively (S2 Fig). We also investigated the possible phylogenetic relationships between YGL-1 and these proteins mentioned above. The results indicated that the proteins from higher plants including monocotyledonous plants (*Z*. mays, *S*. *italica*, *O*. *sativa* and *B*. *distachyon*), dicotyledonous plant (*A*. *thaliana*), bryophyte (*P*. *patens*) and pteridophyte (*S*. *moellendorffii*) are divided clearly into one subgroup, while the proteins from lower plants (*A*. *protothecoides* and *C*. *reinhardtii*) form another subgroup (Fig 5). These data indicate that the YGL-1 (GRMZM2G007441) is conserved in plants.

### Expression analysis of *YGL-1*

**Fig 6:**
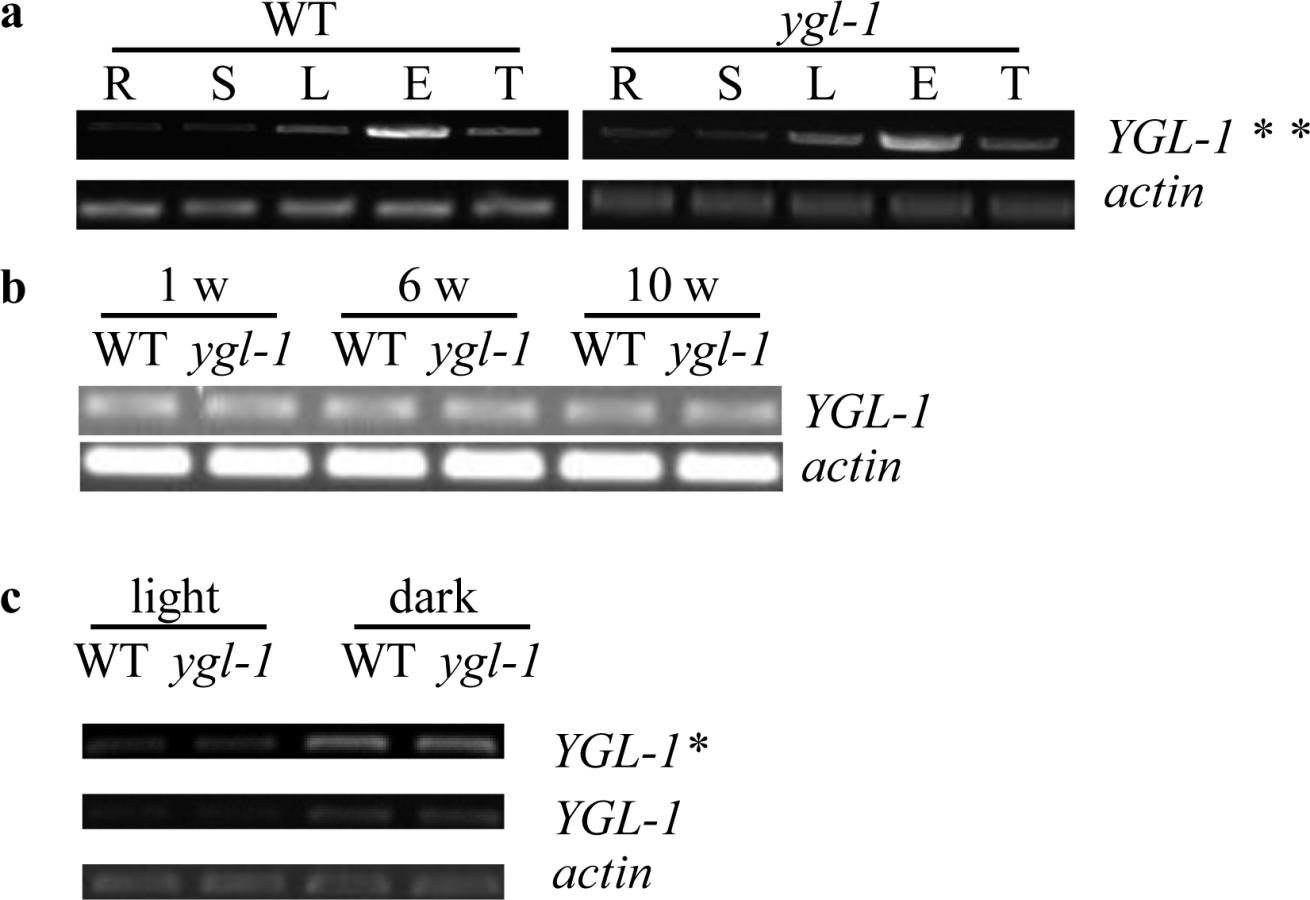
Semi-quantitative RT-PCR analysis of *YGL-1*. (a), Expression profiles of *YGL-1* in root (R), stem (S), leaf (L), ear (E) and tassel (T) of WT and *ygl-1* mutant. (b), *YGL-1* expression in WT and *ygl-1* mutant leaves of 1-, 6- and 10-week-old plants. (c), *YGL-1* expression in WT and *ygl-1* mutant leaves of 1-week-old plants grown under light or dark conditions. *actin* was amplified 24 cycles and *YGL-1* was amplified 28 cycles because the gene expression was too low to be visible when amplified 24 cycles. *, *YGL-1* was amplified 30 cycles; **, *YGL-1* was amplified 32 cycles. WT, wild type plants of Lx7226.

To compare *YGL-1* expression profiles, total RNA was extracted from roots, stems, leaves, ears and tassels of the *ygl-1* mutant and wild-type plants, respectively. Semi-quantitative RT-PCR analysis was performed. *YGL-1* mRNA was expressed in all tested tissues with relatively higher levels in ears than in the other four organs (Fig 6A). Expression levels of the *YGL-1* transcript were similar in the *ygl-1* mutant and wild type from early to mature stages (Fig 6B). These results indicated that the premature termination mutation in the coding region of *ygl-1* did not result in variation of its own mRNA expression. Furthermore, the effect of light and dark growth conditions on the expression of *YGL-1* was conducted. Transcript levels were higher when *ygl-1* or wild-type plants were grown under dark than light conditions, indicating that light intensity regulates the *YGL-1* or *ygl-1* mRNA expression (Fig 6C).

### The effect of the *ygl-1* mutation on the expression of other genes

**Fig 7:**
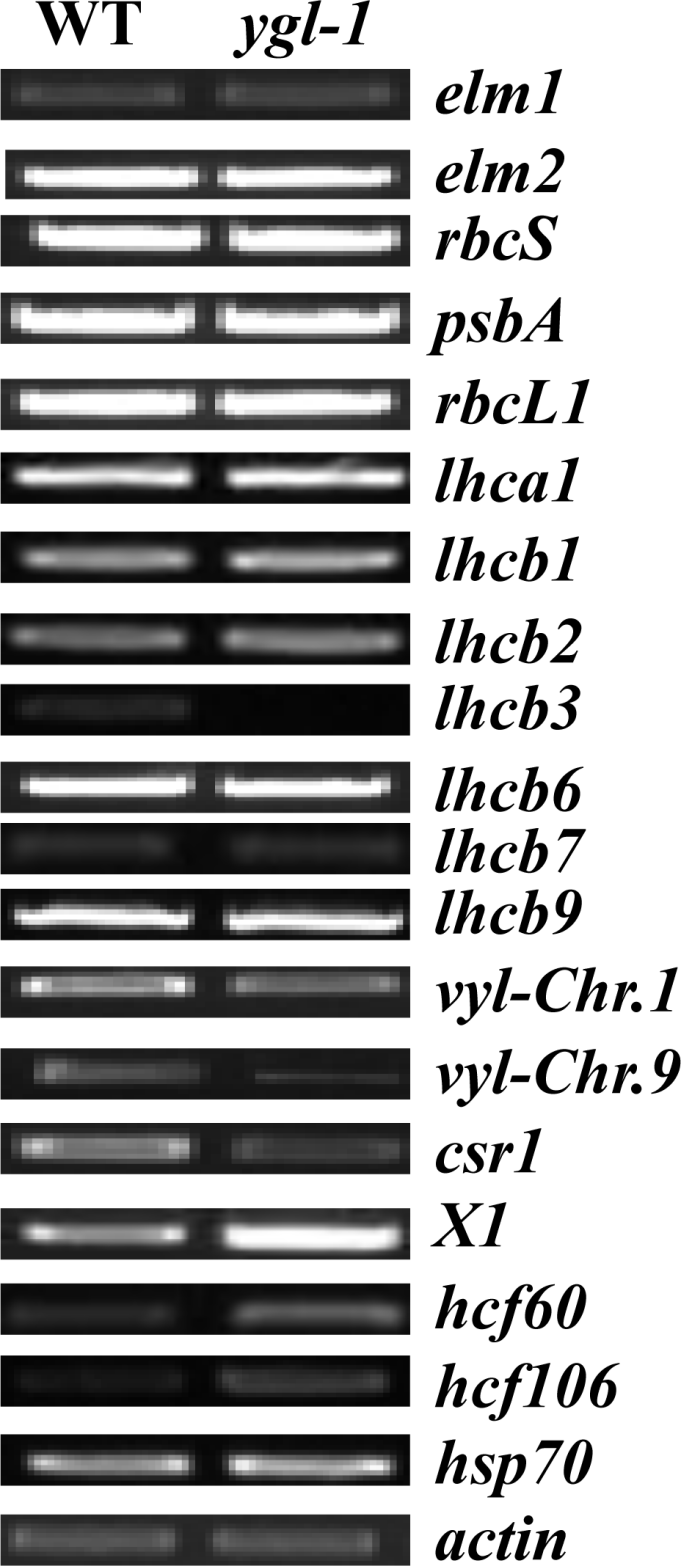
Expression analysis of nineteen genes associated with Chl biosynthesis, photosynthesis or chloroplast development by semi-quantitative RT-PCR analysis. Total RNA was isolated from WT and *ygl-1* leaves of six-week old plants. All these genes were amplified 24 cycles. *actin* was amplified as a control. WT, wild type plants of Lx7226.

To investigate whether the *ygl-1* mutation affected the transcript levels of genes associated with Chl biosynthesis, photosynthesis and chloroplast development, semi-quantitative RT-PCR primers for 19 genes related to these pathways were designed (S1 Table). Semi-quantitative RT-PCR analysis was conducted in six-week old leaves of *ygl-1* and wild type plants. The expression of genes involved in Chl biosynthesis, such as *elongated mesocotyl1* (*elm1*) and *elongated mesocotyl2* (*elm2*), showed no difference between the *ygl-1* mutant and wild type [13–14] (Fig 7). *rbcS* is a nuclear gene encoding the small subunit of Rubisco [40]. *psbA* and *rbcL1* are chloroplast genes encoding reaction center polypeptides and the large subunit of Rubisco [41–42], respectively. Their mRNA levels were also not significantly different between the *ygl-1* mutant and wild type (Fig 7). 7 nuclear genes (*lhca1*, *lhcb1*, *lhcb2*, *lhcb3*, *lhcb6*, *lhcb7* and *lhcb9*), encoding the light-harvesting chlorophyll proteins (LHC-PI or LHC-PII) which are synthesized in the cytoplasm and after import into chloroplast, then they are targeted and inserted into thylakoid membrane, their transcript levels were also compared. 6 genes (*lhca1*, *lhcb1*, *lhcb2*, *lhcb6*, *lhcb7* and *lhcb9*) were at a similar transcript level in the *ygl-1* mutant and wild type (Fig 7). *lhcb3*, encoding the light harvesting chlorophyll *a*/*b* binding protein of PSII, was altered in the *ygl-1* mutant, with no expression or too low to be visible (Fig 7). Interestingly, 6 other genes participating in chloroplast development or function were also significantly different in the *ygl-1* mutant and the wild type. *vyl-Chr*.*1* and *vyl-Chr*.*9*, encoding the ClpP5 subunit of Clp protease which was important for chloroplast development or function [15, 43], were slightly decreased in the *ygl-1* mutant (Fig 7). *csr1* (chloroplast SRP receptor1) encoding cpFtsY important for efficient thylakoid targeting of LHCPs [35], showed severely reduced mRNA level in the *ygl-1* mutant (Fig 7). *X1*, which was predicted to encode *Zea mays* signal recognition particle 54 kDa protein and act as a chloroplast receptor (GenBank), showed significantly increased expression in the *ygl-1* mutant (Fig 7). *hcf60*, a nuclear gene, encodes the chloroplast ribosomal small subunit protein 17 [33]. *hcf106*, another nuclear gene, is required for post-translational steps in the biogenesis of the chloroplast cytochrome b6f complex in maize [44]. Their transcript levels were also significantly increased in the *ygl-1* mutant (Fig 7). *hsp70*, encoding a stromal chaperone protein which facilitates protein folding and plays a role in protein translocation across membranes, did not appear to be significantly affected in *ygl-1* [45] (Fig 7). Overall the *ygl-1* mutation might mainly affect the mRNA levels of some genes involved in chloroplast development but not genes associated with Chl biosynthesis or photosynthesis. These results suggested that the expression of these genes associated with chloroplast development might be regulated at the translational or post-translational levels, since the mutation of *YGL-1* did not alter its own mRNA expression in *ygl-1* (Fig 6A and 6B).

## Discussion

In recent years, researchers have paid increasing attention to leaf-color mutations, with important insights into the mechanisms of these mutations gained by study of several organisms. In maize, the molecular mechanisms of leaf-color mutation and the related responsible loci are still far from being fully understood. Mutant analysis is an effective approach to explore the function of genes in chloroplast development.

### SRP43 confers the yellow-green mutation in *ygl-1*

Taking a positional cloning approach, the *ygl-1* gene was mapped to an interval of 48 kb in bin 1.01 on the short arm of chromosome 1. To our knowledge, only *vyl-Chr*.*1* related to leaf-color mutation has been previously mapped and cloned on the same chromosome [15]. Most homozygous *vyl-Chr*.*1* plants died within 3 weeks of emergence and the surviving plants were pale-yellow and very small. In contrast, homozygous *ygl-1* plants were normal and can grow to maturity. In addition, the *vyl-Chr*.*1* gene is located in bin 1.03, not in the 48 kb region in bin 1.01 of *ygl-1*, so we assert that *ygl-1* is a novel gene conferring leaf-color mutation in maize.

The chloroplast signal recognition particle (cpSRP), composed of one cpSRP43 dimer and one cpSRP54 monomer, is required for binding the light-harvesting chlorophyll protein (LHCP) to form a cpSRP/LHCP transit complex which targets LHCP to the thylakoid membrane [46–47]. Mutants in SRP54 have been isolated in *Arabidopsis* and rice. The mutant *ffc*, which was generated by ethyl methane sulfonate (EMS) in *Arabidopsis*, had yellow first true leaves that subsequently turned pale green [48]. A mutant in SRP54 of rice, designated as *YGL138*(*t*), derived from Nipponbare treated by EMS, showed yellow-green leaf phenotype throughout development [21]. Could a mutant in SRP43 also lead to chlorophyll deficient mutation? The *chaos* mutant of SRP43 in *Arabidopsis*, resulting from insertion of an enhancer trap *Ds* element, showed pale leaves throughout the vegetative cycle [49, 22]. The double mutant *ffc*/*chaos* had pale yellow leaves from early stage to maturity [50]. Mutations in CrcpSRP43 would lead to a low Chls content, high Chl *a*/*b* ratio and leaf-color phenotype in *C*. *reinhardtii* [51]. The two cpSRP43 mutants in *Arabidopsis* and *C*. *reinhardtii* showed a similar and specific decline of LHCPs in the developed chloroplast, indicating that the cpSRP43 protein plays a conserved function in green microalgae and higher plants, which is also supported by our phylogenic analysis. To our knowledge, no mutant in SRP43 has yet been reported in model monocotyledonous plants including rice and maize. We postulated that mutation in cpSRP43 of maize will result in low levels of Chls and Car, higher Chl *a*/*b* ratio and a leaf-color phenotype. In our study, a leaf-color mutant *ygl-1* was isolated and it developed yellow-green leaves at all stages. The *ygl-1* gene was fine mapped to a 48 kb region in the maize genome. Three candidate genes were identified in the 48 kb chromosomal region of *ygl-1*. One of these, *GRMZM2G007441*, was predicted to encode a cpSRP43 protein and act as a chloroplast precursor. We suspect that some mutational site in the *GRMZM2G007441* genomic sequence which leads to mutation in the encoded product exists in *ygl-1*. Sequencing results revealed that 1-bp was deleted in the coding region of this gene in *ygl-1*, and the single nucleotide deletion led to a frame shift mutation. *ygl-1* mutants showed significantly reduced contents of Chls, Car, and higher Chl *a*/*b* ratios than wild-type plants at both seedling and tasseling stages. We presumably assume that the defect in *ygl-1* lead to the chlorophyll deficiency in the *ygl-1* mutant, which makes it to exhibit yellow-green leaf phenotype throughout the growth period. In addition, higher Chl *a*/*b* ratios in *ygl-1* might make the significantly decreased content of Chl *b* to play an important role in its yellow-green leaf phenotype. These results provided further evidence that *GRMZM2G007441* was the gene responsible for *ygl-1* in maize.

### SRP43 is an important component of the SRP protein and plays a central role during chloroplast development

The cpSRP is a collection of four proteins that work together: cpSRP54, cpSRP43, cpFtsY and ALB3 [36]. These SRP proteins are believed to be involved in the proper folding of the LHCP_S_ and their targeting to the thylakoid membrane [52]. In the chloroplast, cpSRP uses two mechanisms to target both chloroplast- and nuclear- encoded LHCP_S_ to the thylakoid membrane [53]. The first pathway was called co-translational targeting. This system, which targets chloroplast-encoded proteins to the thylakoid membrane, requires cpSRP54 and the conserved SRP RNA of the bacterial and cytosolic ribonucleoprotein SRPs. The second pathway called post-translational targeting, targets nuclear-encoded proteins to the thylakoid membrane using cpSRP54 together with cpSRP43, but lacks the conserved SRP RNA of the bacterial and cytosolic ribonucleoprotein SRPs.

In the latter targeting pathway, the cpSRP43 works as a novel chaperone specific for light-harvesting chlorophyll *a*, *b*-binding proteins and its role cannot be taken over by other stromal chaperones [54–55]. This protein alone is sufficient to form a soluble complex with LHCPs and to prevent aggregation [54]. This supports the idea that cpSRP43 alone is sufficient to targeting of LHCPs to the thylakoid membrane [56]. The cpSRP43 protein is also able to dissolve aggregated LHCPs [54]. This ability of cpSRP43 might be advantageous if LHCP import exceeds the capacity of cpSRP43 to form the transit complex.

The cpSRP43 consists of ANK repeat domains and CHROMO domains [54]. The cpSRP43 ANK repeat domains provide the binding site for the conserved DPLG motif in LHCPs and are sufficient for the chaperone function, whereas the chromo domains are dispensable. In our study, the cpSRP43 protein was 319 amino acids long in the *ygl-1* mutant, in which the ANK repeat domains changed a lot and the CHROMO domain almost disappeared due to the 1-bp deletion in the coding region which led to a premature termination codon. We hypothesize that the deficiency chlorophyll, the impaired development of chloroplasts, the decreased capacity of photosynthesis and the yellow-green leaf phenotype of *ygl-1* might be due to the changed ANK repeat domains of cpSRP43, which might be incapable of binding the conserved DPLG motif in LHCPs to function as chaperone correctly. In our semi-quantitative RT-PCR results, *lhcb3*, encoding light harvesting chlorophyll *a*/*b* binding protein of PSII, was specifically suppressed in *ygl-1* plants compared with the wild-type plants, whereas all other components of PSI (lhca1) and PSII (lhcb1, lhcb2, lhcb6, lhcb7 and lhcb9) remained unchanged. These findings indicate that at least lhcb3 is dependent on the cpSRP43 for targetting, other 6 proteins are either less dependent or independent of cpSRP43. This observation supports the idea that mutations in cpSRP have specific effects on a subset of LHCPs [48]. *hcf106*, encoding an integral membrane protein which is required in △pH pathway for targeting proteins to the thyakoid membranes [44, 57–59], showed significantly increased expression in the *ygl-1* mutant. The increase in *hcf106* might occur in response to the defect of functional cpSRP43. *hsp70*, a nuclear gene encoding a stromal chaperone protein [45], remained a similar transcript level in *ygl-1* and wild type. This result is in consistent with previous reports in *Arabidopsis ffc* and *chaos* mutants [48]. In addition, the stable level of some gene transcripts (participating in Chl biosynthesis such as *elm1* and *elm2*, or photosynthesis such as *rbcS*, *psbA* and *rbcL1*) and the differently affected level of other gene transcripts (involving in chloroplast development such as *vyl-Chr*.*1*, *vyl-Chr*.*9*, *csr1*, *X1* and *hcf*60), probably caused by a feedback mechanism, suggest that *ygl-1* mainly altered the transcript levels of genes involved in chloroplast development. The *ygl-1* mutant had less non-appressed granal stacks and rare and less dense granal stacks at the seedling and tasseling stages, respectively. The suppressed development of chloroplasts and yellow-green leaf phenotype in the *ygl-1* mutant is likely due to the apparent up/down-regulations of genes (*hcf106*, *vyl-Chr*.*1*, *vyl-Chr*.*9*, *csr1*, *X1* and *hcf*60) that participated in chloroplast development, which response to the defect of *ygl-1* function that are responsible for the normal chloroplast development. However, transcript levels of genes that involved in Chl biosynthesis and photosynthesis were stable, which makes why the contents of Chls and Car were reduced and the capacity of photosynthesis was decreased is still not very clear.

In the higher plant post-translational pathway, the cpSRP43 binds to the imported LHCPs, then cpSRP54 binds to the cpSRP43-LHC protein complex. This complex subsequently binds to a membrane-bound cpFtsY, and finally this LHC-cpSRP43-cpSRP54-cpFtsY complex is guided to the ALB3 protein [34]. In the co-translational pathway, cpSRP54 binds to a chloroplast-encoded protein that is newly synthesized by a ribosome, then cpFtsY guides this complex to the ALB3 protein that works in tandem with cpFesY. Our semi-quantitative RT-PCR results revealed that *csr1*, which encodes cpFtsY [35], showed severely suppressed transcript level in the *ygl-1* mutant. *X1*, which was predicted to encode cpSRP54 and act as a chloroplast receptor (GenBank) showed significantly increased expression in the *ygl-1* mutant. These results suggest that there might be alternative explanations for the respective interactions of these genes with cpSRP43. In addition, the *ygl-1* mutant does not show a developmental effect on chlorophyll content, chloroplast ultrastructure, or yellow-green leaf phenotype. The transcript level of *YGL-1* remained at a similar level from 1-week to 10-week stages both in *ygl-1* and wild type, indicating that the requirement for SRP43 is not dependent on plant age. Taken together, we suggest that the cpSRP43 protein plays a central role in the post-translational targeting pathway during chloroplast development.

## Conclusion

In this study, a novel yellow-green leaf mutant *ygl-1* was isolated from maize. The mutant spontaneously showed a distinguishable yellow-green leaf phenotype throughout the growing period and was controlled by a single recessive nuclear gene. The *YGL-1* locus was delimited within a 48 kb region in the maize genome harboring three candidate genes. A single base pair deletion discovered in cpSRP43 might lead to reduced contents of Chls and Car, an increased ratio of Chl *a*/*b*, defective chloroplast development and declined capacity of photosynthesis, which conferred the yellow-green character in *ygl-1*. The mutation in *ygl-1* might regulate the expression levels of some genes associated with chloroplast development at translational or post-translational levels, while its own mRNA level was not affected. Our results are a valuable foundation for further study and functional characterization of *ygl-1*, which will facilitate dissection of the mechanism of cpSRP43 protein involvement in chloroplast development of maize.

## Supporting Information

S1 Fig Amino acid sequence alignment of *ygl-1* and six wild-type inbred lines. DNAMAN 5.0 software was used for sequence alignment. The light blue and red lines represent ANK_2 and ANK region, respectively, and the dark blue line represents CHROMO region.
(TIF)

S2 Fig Amino acid sequence alignment of the YGL-1 protein and its homologues. The light blue and red lines represent ANK_2 and ANK region in YGL-1, respectively, and the dark blue line represents CHROMO region in YGL-1. Accession numbers of the protein sequence were as follows: *Zea mays* [YGL-1, DAA43190.1]; *Sorghum bicolor* [XP_002465920.1]; *Setaria italica* [XP_004985832.1]; *Oryza sativa* [NP_001048866.1]; *Brachypodium distachyon* [XP_003562136.1]; *Arabidopsis thaliana* [NP_566101]; *Physcomitrella patens* [XP_001771412.1]; *Selaginella moellendorffii* [XP_002989974.1]; *Auxenochlorella protothecoides* [XP_011399198.1]; *Chlamydomonas reinhardtii* [AGC59877.1]. DNAMAN software was used for the sequence alignment.
(TIF)

S1 Table Semi-quantitative RT-PCR primers. (DOCX)

S2 Table Gene annotation within the located region. (DOCX)
