# Supplementary figures and images for: Fine Mapping and Candidate Gene Analysis of the Leaf-Color Gene ygl-1 in Maize
Source: PLoS One. 2016 Apr 21;11(4):e0153962. doi: 10.1371/journal.pone.0153962 (PMC4839758; doi:10.1371/journal.pone.0153962)

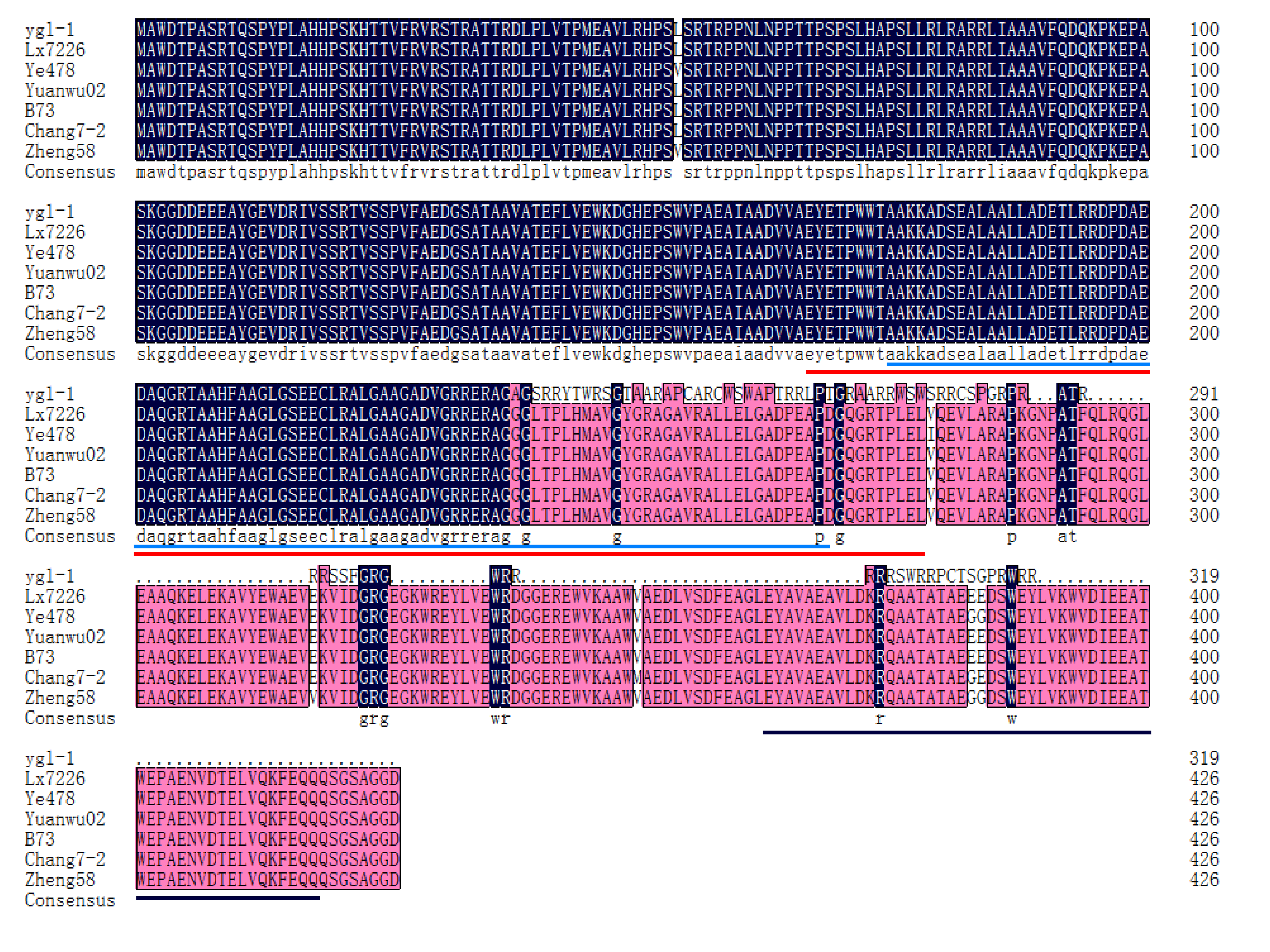

Supplement: S1 Fig — DNAMAN 5.0 software was used for sequence alignment. The light blue and red lines represent ANK_2 and ANK region, respectively, and the dark blue line represents CHROMO region. (TIF) [file pone.0153962.s001.tif]
