# Supplementary material for: Fine Mapping and Candidate Gene Analysis of the Leaf-Color Gene ygl-1 in Maize
Source: PLoS One. 2016 Apr 21;11(4):e0153962. doi: 10.1371/journal.pone.0153962 (PMC4839758; doi:10.1371/journal.pone.0153962)
Supplement: S1 Table — (DOCX) [file pone.0153962.s003.docx]

**S1 Table. Semi-quantitative RT-PCR primers.**

| **Gene** | **Gene ID or accession** | **primer sequence (5′-3′)** | **Product size (bp)** |
| --- | --- | --- | --- |
| *elm1* | DR830444.1 | F:GCCTTGTCATTCAGCACTTCC | 303 |
|  |  | R:TCTCAGCGACTCACTTGTAATCTTG |  |
| *elm2* | GRMZM2G101004 | F:TCCGCTGTCCCGTGGTATG | 222 |
|  |  | R:GCCTCCAGCAGTATGAGCAAAG |  |
| *rbcS** |  | F:CAACAAGAAGTTCGAGACG | 259 |
|  |  | R:CGGGTAGGATTTGATGGC |  |
| *psbA* | AF543684.1 | F:CTTCATCGCTGCTCCTCC | 241 |
|  |  | R:CCACTCACGACCCATATAACA |  |
| *rbcL1* | V00171.1 | F:AACTTTCCAAGGTCCGCCAC | 269 |
|  |  | R:TGATTTCACCAGTTTCGGCTTG |  |
| *lhca1* | GRMZM2G038519 | F:GGCGACTTCGGCTTTGAT | 437 |
|  |  | R:GCGAGGCGTCCGTTCTT |  |
| *lhcb1* | GRMZM2G351977 | F:GCGTGCCAGGTGGTGCTC | 284 |
|  |  | R:GCCCAGGCGTTGTTGTTGA |  |
| *lhcb2* | AC207722.2_FG009 | F:AGGTCGTGCTCATGGGTG | 271 |
|  |  | R:GCGTTGTTGTTGACTGGGT |  |
| *lhcb3* | GRMZM2G155216 | F:TCGGGTTCTTCGTCCAGG | 365 |
|  |  | R:CAACGCACGGGCATTCA |  |
| *lhcb6* | GRMZM2G018627 | F:ACCAGACCACCAGCTTCCTC | 391 |
|  |  | R:CCGCCCTCCGAGAAGAT |  |
| *lhcb7* | GRMZM2G402936 | F:TGTTTCCCTCCGTCGCTTTA | 604 |
|  |  | R:GCCCAGATGGCAAGGATG |  |
| *lhcb9* | GRMZM2G018627 | F:CAGCAAGACCAACAAACCCA | 486 |
|  |  | R:CCGCCCTCCGAGAAGAT |  |
| *vyl-Chr.1* | GRMZM2G056373 | F:GAGCGGTTCCAGAGCGTCG | 204 |
|  |  | R:GCCATACTCCTTTGCTTCTTTTGC |  |
| *vyl-Chr.9* | GRMZM2G121456 | F:CGGTTCCAGAGCGTCGTC | 506 |
|  |  | R:CCATACTCCTTTGCTTCTTTCG |  |
| *csr1* | AJ549215.1 | F:GCAGTTCTTTCGCAGGCAGTA | 307 |
|  |  | R:GCTCATCCACAACGCTAACCA |  |
| *X1* | XM_008681195.1 | F:GGGAGCGATTAGAGGACCTTG | 456 |
|  |  | R:GCATCTGAGCACGCATTTGG |  |
| *hcf60* | Y19204.1 | F:CCGAGACAGGAGAAGGCAAG | 334 |
|  |  | R:GCGGGATGGCGAGGAA |  |
| *hcf106* | GRMZM5G898735 | F:TCTTTCGTTGCTGTAGGGAG | 302 |
|  |  | R:GGCAGGCTGTTGTGGATT |  |
| *hsp70* | GRMZM2G340251 | F:AACCAGGTCGCCATGAAC | 301 |
|  |  | R:GCCTCTGTGAGTCATTGAAGTAG |  |
| *actin* | GRMZM2G126010 | F:TCACCCTGTGCTGCTGACCG | 191 |
|  |  | R:GAACCGTGTGGCTCACACCA |  |

*This primer came from previous study [31].
