# Supplementary material for: Fine Mapping and Candidate Gene Analysis of the Leaf-Color Gene ygl-1 in Maize
Source: PLoS One. 2016 Apr 21;11(4):e0153962. doi: 10.1371/journal.pone.0153962 (PMC4839758; doi:10.1371/journal.pone.0153962)
Supplement: S2 Table — (DOCX) [file pone.0153962.s004.docx]

**S2 Table. Gene annotation within the located region.**

| **Transcript ID** | **Physical position on Chr.1** | **Protein length (aa)** | **Predictive Function** |
| --- | --- | --- | --- |
| GRMZM2G071349 | 7302139-7302908 | 146 | Vitamin K epoxide reductase |
| GRMZM2G007441 | 7313312-7314961 | 426 | probable signal reconition particle 43kDa protein, chloroplastic-like |
| GRMZM2G096020 | 7341979-7348599 | 551 | putative AMP-dependent synthetase and ligase superfamily protein |
